# The London Clinical Congress

**Published:** 1914-05-16

**Authors:** 


### The London Clinical Congress.

From July 27 to August 1 the London Session of the Clinical Congress of Surgeons will be held. The days will be spent in visiting the theatres of the various hospitals, where demonstrations by distinguished surgeons will be witnessed, and during the evenings addresses will be delivered and papers read by surgeons from various parts of the world. The General Surgical Division will meet in the Grand Hall of the Hôtel Cecil, and the Division of Surgical Specialities in the Ballroom of the Savoy. On Monday, July 27, Sir R. J. Godlee, P.R.C.S., will give an address of welcome, and the inauguration of the new president, Dr. J. B. Murphy, will take place after the retiring president, Dr. G. E. Brewer, has delivered an oration. Dr. Charles H. Mayo, Rochester, U.S.A., whose renowned institution was described in The Hospital of April 19, 1913, p. 73, is to be one of the readers of the papers, having chosen for his subject "Primary and Late Results of Operations for Exophthalmic Goitre or Hyperthyroidism"; while Sir Arbuthnot Lane will start a discussion on the papers concerning "Intestinal Stasis" which are to be read by Dr. J. C. Bloodgood and Sir William Osler. The general secretary of the congress is Dr. Franklin H. Martin, M.D., 1 Wimpole Street, W. The London Committee consists of Sir Rickman J. Godlee, hon. chairman, and Mr. Herbert J. Paterson, F.R.C.S., and Mr. Herbert S. Pendlebury, F.R.C.S., hon. secretaries.

Lord Lamington, G.C.M.G., G.C.I.E., has consented to be President of the Research Defence Society, in succession to the late Sir David Gill, K.C.B., F.R.S.

